# Bodies sensing air pollution in asthma research

**DOI:** 10.1080/09505431.2025.2491342

**Published:** 2025-05-05

**Authors:** Emma Garnett

**Affiliations:** Geography Department, University of Exeter, Exeter, UK

**Keywords:** Air pollution, asthma, sensing, knowing bodies, participatory research

## Abstract

Air pollution knowledge practices are rapidly changing in science and policy research because of growing awareness of its harmful effects on environmental and human health. Informed by developments in environmental epigenetics and exposomics, a turn to the body is now evident, which includes generating more granular data to define individual disease risks and specify personal health interventions. By turning their attention towards the body, scientific researchers are also zeroing in on the contexts and conditions of exposure, and how living environments impact air’s toxicity. Evidence shows air pollution can contribute to the development of asthma. It is not just episodes of high levels of air pollution that matter but its sources, the timing of exposure, and accumulative effects. The knowledge practices of air pollution science are therefore useful sites for exploring how air pollution is entangled within the powerful systems and political economies that enact it. The recent monitoring of asthmatic bodies (rather than environments) with wearable sensors are informing knowledge about how and when exposure happens and is particularly harmful. Yet this more contextual, practical knowledge is often disregarded because the primary focus is on generating more data. By centring the ‘sensing body’  – which highlights the spaces, times and social practices of exposure that contribute to and worsen asthma  – scientific studies can generate more critical analyses of air pollution that are relevant for guiding actions to prevent harm.

## Introduction

London Mayor Sadiq Khan has referred to air quality in some areas of the city as the worst since modern records began. Although materially and chemically unstable, the standard procedures of in-real-time monitoring constructs air pollution as a scalable, knowable, and data-centric problem. This continuous generation of data creates digital archives of air pollution that serve a modern political imagination, in which environmental phenomena are uniform and comparable with one another (Barry, 2001). Employed in environmental regulation to demonstrate legal compliance to air quality standards and to support public health knowledge and surveillance, the vast data archives of air pollution inform an atmospheric and spatial government with science (Whitehead, 2009).

There is growing recognition within science and policy communities globally that these ways of knowing polluted atmospheres are insufficient for protecting health (Troeger *et al.*, 2018). Environmental data used in regulation measure single air pollutants at fixed spatio-temporal points even though atmospheric fluctuations and chemical reactions mean air is always transforming. Evidence also shows that for most air pollutants there are no safe limits (Wei *et al.*, 2024). Even at low levels, and particularly in early life, air pollution can alter gene-expression and future health trajectories. This indeterminacy of air pollution and its long-term health effects is encapsulated in the problem of rising rates of asthma globally. Evidence consistently shows a short-term association between exposure to air pollution and asthmatic episodes, but the accumulative, latent effects of air pollution on the development of asthma are far more difficult to discern.

In response, the interdisciplinary field of air pollution science is zeroing in on the body as a site for researching how and when exposures influence health outcomes. New technologies in environmental toxicology and molecular epidemiology can track exposures over time, turning bodies into sensors for identifying the precise mechanisms of air pollution-related disease (Fortun, 2011). In the life sciences, the identification of epigenetic alterations like DNA methylation and oxidative stress are helping to explain the different contexts and causes of asthma development in individuals. The ‘human exposome’ project, for instance, promises to overhaul conventional risk assessments by addressing the complex and uneven interplay of multiple exposures over the life course (e.g. Vineis *et al.*, 2017). In this work, emphasis is on the non-genetic drivers of health and disease, and therefore the complex chemical processes of toxicants and wider environmental factors that exert pressure on our bodies and health.

Research in Science and Technology Studies (STS) is also engaging in discussions of how environmental pollution and chemical exposure demand different kinds of knowledge and responses (Fortun, 2014; Liboiron *et al.*, 2018). Researchers are highlighting the challenges and limits of integrating everyday experiences of environmental pollution with the standards and thresholds used in policy (Ottinger, 2009; Gabrys *et al.*, 2016). There has been a particular focus on sensory ways of knowing pollution and toxicity that lie beyond evidentiary data and evidence practices (Shapiro, 2015; Calvillo and Garnett, 2019). However, there has been less emphasis on how scientists working in this field engage with the human, embodied and material dimensions of pollution in health research and policy (see Muñoz Duque *et al.*, 2024). And in particular how, by drawing attention to living environments, these new ways of knowing pollution are raising important questions about how bodies and environments come to be in relation in particular (often uneven) ways (Braun, 2007; Murphy, 2008; Meloni *et al.*, 2021).

This study contributes to STS traditions that conceptualise pollution as an assemblage of material effects  – entangling the human body within powerful systems and political economies that enact it (Murphy, 2008; Roberts, 2017; Liboiron, 2021). It also builds on a growing body of literature examining how advances in the life sciences, such as epigenetics and exposomics, illuminate the role of social and material environments in shaping health and its causes (Landecker, 2013; 2015; Guthman and Mansfield, 2012; Meloni, 2019; Meloni *et al.*, 2021; Lamoreaux, 2023). The empirical sections of the article review how scientific and medical advancements shape understandings of the link between air pollution and asthma, extending both these areas of research. Through discourse analysis of science-policy documents and interviews with scientists in this field, the study investigates how the entanglements of bodies and air influence data, knowledge practices, responses, and the allocation of responsibility. Drawing on these insights, I ask: what role does the human body play in research on air pollution and health? What are the possibilities (and limitations) of knowing air pollution in, through and with the body in the context of environmental health knowledge and politics? How may these perspectives offer new ways of knowing, responding, and living with air pollution amidst multiple, compounding exposures?

To address these questions, I begin by developing my analytical perspective through a synthesis of STS and social science scholarship to explore the chemical entanglements of bodies and environments and the production of knowledge about disease. After briefly outlining methods, I use my empirical analysis to elaborate how the body plays a crucial role in shaping data and informing evidence practices in air pollution science. Specifically, I highlight how the material and chemical exchanges that define bodies and environments enable scientists to detect and track the movement and variations of air across urban environments and over time. By emphasising the sensory capacities of bodies in knowledge of exposure and disease, I argue that a more expansive understanding and approach to the body in air pollution science could drive a stronger sense of responsibility for its complex causes and individual harms, grounded in more critical forms of knowledge.

## Analytical perspective

Recent developments in the Anthropocene and postgenomic science mean it is now ‘untenable to consider health as an individual property limited to the space of one’s body or the time of one’s lifespan – or even as something wholly human’ (Ford, 2019, no page number). Changing models of health are emerging in response to evidence of the ways human activities imprint upon and transform micro-biological processes, for instance in the growing field of environmental epigenetics (Meloni *et al.*, 2021; Rose *et al.*, 2022). This article engages with analytical perspectives in STS that examine how polluted environments ‘alter’ bodies and what this means for knowledge production about its causes and ways of living with it (Murphy, 2017). Despite growing awareness of the social and political systems that produce pollution and disease, science and policy research continue to inform individualised accounts of exposure, vulnerability and mitigation. For the purposes of this article, where I wish to rethink exposed bodies as active participants rather than objects in knowledge production, I focus on analytical perspectives that discuss bodies in relation to (1) chemical ecologies, (2) embodied sensing and (3) knowledge practices.

### Chemical ecologies

Discussing work on environmental epigenetics, Guthman and Mansfield (2012) put forward an ecological model of the body to draw attention to how the external environment becomes part of the body and how the body is part of its own remaking (see also Landecker, 2015). They draw on their respective research of environmental epigenetics to emphasise how studies of the health effects of ‘xenobiotic’ chemicals fundamentally challenge standard models of the relationship between environments, bodies, and health:

spatial epidemiology assumes a static relationship between space and population. Snapshots are taken to establish correlative relationships, and the existence of a spatial cluster of a disease or condition demonstrates an exposure. Yet, just as chemicals move in and out of bodies, they move in and out of particular spaces. People are mobile as well; those exposed elsewhere may reveal diseases not from those spaces, while those in those spaces might not be manifesting the disease (Guthman and Mansfield, 2012, p. 498).

The assumption that chemical pollutants can be monitored and tracked in space and time, including the atmosphere, denies their material, chemical volatility and the traffic of biochemical pathways. Indeed, and as these authors also write, substances flow through environments and bodies, interacting in unpredictable, often subtle ways.

The dynamic fluctuations of chemicals in and out of bodies brings to the fore the complex spatialities and temporalities of health. In this view the body is porous, receptive, and reactive; it is composed by experience and history rather than just biology.

Vanessa Agard-Jones (2013) and Michelle Murphy have discussed how the embodied experiences of pollution and exposure are ‘entangled and enfolded’ in the ‘political economy of chemical relations’ (Murphy, 2008, p. 701). Arguing that bodies matter in the world, these authors analyse the body and ‘the chemicals circulating within and beyond it’ to generate insight into ‘how individual bodies and individual people come to be, in dynamic relationship to the worlds around them’ (2013, p. 192). Taking a chlordécone molecule (emitted in pesticides) as their primary unit of analysis, Agard-Jones shows how bodies in postcolonial Martinique are ‘connected to commodity chains, to uneven relations of colonial/postcolonial power, and thus to world systems’ (2013, p. 192).

As Murphy (2008) has similarly argued, these chemically altered bodies and environments highlight ‘chemical regimes of living’ that are difficult to calculate in terms of individual risk and through linear causal chains. Using archival methods, Murphy shows how relations between economy and population have been historically and materially assembled and, in doing so, demonstrates the situatedness and contingencies of these assemblages. Murphy suggests that recognising these altered states can compel us to confront the pervasive chemical violence embedded in capitalist and colonial infrastructures. Moreover, it invites an envisioning of alternative ways of living that resist apocalyptic narratives or reductive accounts of individual exposure and responsibility (see Rose, 2007; Braun, 2007).

### Sensing bodies

The idea of the body as a site of chemical alteration and therefore figure of contamination and harm is not new. The canary in the coalmine is perhaps the most well-known warning figure of polluted atmospheres in an age of extraction at the onset of industrialisation in the nineteenth century. In a more recent example, Hannah Landecker (2013) refers to the experimental rodent in twentieth century life science as a sentinel for the legacies of industrialisation after the accidental discovery of the endocrine disrupting effects of Bisphenol A. Studying the biology of infertility in the 1990s using mouse strains that have high numbers of ‘chromosomal abnormalities’, the biologist Patricia Hunt found that her ‘healthy’ control animals also started showing signs of aberrant cell division. After a systematic review of the handling of the mice over the previous weeks, Hunt’s team determined it was the plastic cages and water bottles sterilised at high heat that caused polycarbonate plastic to leach into rodent bodies. These chemical reactions signalled the toxic embodiment of consumer materials ubiquitous in Western environments.

The sensitivity and responsiveness of bodies to their living environments means they can serve as indicators of often imperceptible or difficult to articulate biological, chemical and environmental dynamics and relations (see also Kelly, 2013; Gramaglia and Mélard, 2019). As Hannah Landecker writes in her discussion of the influence of the ‘environmental turn’ in epigenetics on the work of hormone biologists, the outside world can be found ‘deep inside the molecular biology of the cell’ (2016, p. 106):

Human social interactions and material cultures are increasingly understood as biologically consequent environmental signals. Within the explanatory framework of epigenetics, such signals become biologically inscribed when transduced into bodies as persistent patterns of molecular conformation.

This is an important shift in the role of the environmental signal, which is received and imprinted on Chromatin (a mixture of DNA and proteins, which form chromosomes in the nucleus of the cell of human organisms). As Landecker writes, the sugar that is eaten ‘is transduced to become the nucleosome structure of chromatin’ (2016, p. 93). This modification and biochemical alteration of the body means ‘social things’ like the food industry become biological templates for future health.

Taking inspiration from Landecker’s work, philosopher Samantha Frost describes epigenetic processes as ‘an interpretive link between the world as a body experiences it and the molecular activity of cells’ because ‘they interiorize a body’s experiences of the world’ and ‘alert a body to its relation to the world’ (2021, p. 22). Following Sociologist Charles Pearce, Frost states that bodily sensitivity is ‘an indexical source of information about the impact of anthropogenic events’ (Meloni *et al.*, 2021, p. 6 cf. Frost, 2021). Frost emphasises how epigenic processes do not express causality, but highlight ‘a material intimacy or causal connection’ to features of social and material environments (Frost, 2021, p. 22).

epigenetic processes are a response to a body’s response to an environmental provocation; they entail that we conceive of bodies as changeable and changing in fundamental ways through their engagement with the lived world (Frost, 2021, p. 19)

As Michelle Jamieson (2017) has similarly argued in her discussion of scientific and clinical evidence of the role of immunoglobulin E (IgE) in the causes of asthma, the changeability and indeterminacy of disease trajectories requires a relational understanding of causality, that includes living conditions and circumstances (on indeterminacy in disease causality see Hinchliffe, 2001; Schrader, 2010). In this literature, the body is shown to be both a sensor of environmental injury and also transformed in ways that means it says something about these environments  – they evince a ‘knowingness’, as Frost puts it.

The political and policy potential of this shift in understanding the sensory body as materially involved in the detection of, and knowledge production about, pollution and exposure is helpfully elaborated by Steve Hinchliffe (2016), albeit in relation to a very different topic. Writing about the United Kingdom’s response to avian influenza, Hinchliffe describes how the viral exchanges of birds migrating made surveillance techniques for controlling the pandemic inadequate. Instead, ‘the gentle science of ornithology took on a strategic urgency’ in which birds were not treated as mute objects of knowledge but as knowledgeable (Hinchliffe, 2016, p. 153). Rather than just counting birds, scientists learned ‘to sense individual birds and record their movements and social relations (their pairings, partings and battles)’ as well as make ‘observations of health, status and changes in demeanour’ (Hinchliffe, 2016, p. 162). For Hinchliffe, this bodily ‘sensorium’ is a way of challenging conventional notions of objectivity and agency, offering new ways of thinking about sensing and knowing (Hayward, 2010, 2012).

### More-than-human knowledge practices

In the work outlined above, the body is not merely a passive recipient of knowledge but an active participant in its production. Hinchliffe’s (2016) work complicates biopolitical arguments that monitoring and surveillance lead to human mastery, showing how birds, as environmental sentinels, resist and challenge dominant epistemic structures. Their bodies are not neutral data points but active contributors to a more expansive understanding of exposure and disease, that incorporates human and non-human entanglements. This shift in perspective is particularly crucial when thinking about environmental exposures that unfold in time and space, and are difficult to measure and monitor using scientific methods (Meloni *et al.*, 2021). Technological sensing devices, such as pollution monitors, increasingly mediate knowledge of air quality in ways that account for differences in exposure (Gabrys, 2019), yet critical scholarship highlights how these tools often privilege the priorities of policymakers and corporations over embodied, lived experiences (Shapiro and Kirksey, 2017). As Liboiron *et al.* (2018) argue, the ‘evidentiary practices’ that characterise efforts to generate data about pollution and its effects erase the relational and social systems that define human life.

In response, alternative research methodologies position bodies as integral to the sensing infrastructure of environmental knowledge. Ethnographers researching the embodied experiences of chemical exposure have detailed how material transformations in the body are integral to intellectual processes of molecular detection (Shapiro, 2015; Kenner, 2018; Jarowski, 2022; Spackman and Burlingame, 2018). Nick Shapiro (2015) describes sensitivities to environmental changes in people concerned about formaldehyde exposure in the USA as ‘bodily knowledge’ and the way people make sense of them a process of ‘bodily reasoning’. For Shapiro’s interlocutors, the bodily detection of formaldehyde in homes is not just informative about domestic air quality but emblematic of the powerful systems that result in social precarity and abandonment. Alison Kenner’s (2021) research on asthma similarly draws attention to how people with asthma use chemical and physical alterations in their bodies to navigate everyday environments in highly situated, relational and affective ways.

Collaborating on Calvillo’s public infrastructure for sensing air pollution, Calvillo and Garnett (2019) also foreground the embodied, collective, and relational dimensions of sensing, demonstrating how bodily processes – touch, breath, ingestion – constitute what they term ‘molecular intimacies.’ These intimate relations challenge static representations of pollution by foregrounding how bodies actively detect, respond to, and engage with their environments in urban settings.

These ethnographic and participatory approaches to sensing emphasise how paying attention to the chemical exchanges of bodies and air can enhance the process of ‘learning to be affected’ by it, by attuning to biological and environmental processes (Despret, 2004). Vincanne Despret (2004) highlights how experiments often fail when researchers treat animals as stable objects of research rather than engaging in responsive relationships with their nonhuman subjects. In the context of participatory data practices in human health research, Deborah Lupton argues for moving away from a cognitive, technical understanding the body to make room for ‘the emplaced, sensory body in communicative and pedagogical activities’ (2018, p. 3). This involves both examining how human flesh is ‘disaggregated and calibrated’ to generate knowledge about human health, as well as devoting attention to the ‘everyday visceral experiences’ of bodies (Lupton, 2018, p. 9 citing Coole, 2013, p. 465). Researching bodies in this open-ended way can illuminate the workings of power in, through and with human bodies, as well as consider the ways participatory approaches to research can assume importance and significance in people’s lives (Lupton, 2018, p. 10).

If bodies know, as Hinchliffe and Frost argue, or ‘think’ as Martin Savranksy (2019) has put it elsewhere, the individual participant that participatory medicine and public health appears to imagine becomes ‘unruly', because how bodies respond to their environments cannot be predetermined. It also adds flesh and substance to the critique made by STS scholars, that attempts to involve people’s lived experiences in knowledge practices can end up ‘devolving responsibilities and costs to individuals’ to manage their own health (Prainsack, 2014). In other words, incorporating the sensory, embodied and material dimensions of bodies in research means it becomes trickier to approach human participants as atomised beings or individuals. Instead, the bodily sensorium of air pollution amplifies other sensory, bodily and affective registers that are relevant for approaching research subjects and lived experience in less prescriptive ways (Whatmore, 2006, p. 607).

### Material and methods

Asthma is not just an empirical concern in this article but informs its methodological approach. The empirical material was produced by tracing how research and evidence of the role air pollution plays in asthma is produced in trans-national air pollution science. This includes an analysis of meeting minutes and reports published by the UK’s Committee for Medical Effects of Air Pollutants (COMEAP), a group of scientific experts who provide advice to the UK government. Its archive is open access, and documents are available to download from the government webpage and The National Archives website.^1^ Ten interviews were also conducted with researchers involved in projects funded by the UK that monitor exposure to air pollution in bodies with asthma. Interviewees include researchers involved in international projects studying exposure in India. In cities in India, cases of asthma are often high and socio-geographical differences cannot be explained using epidemiological evidence that is largely based on research conducted in the Global North (Negi and Srigyan, 2021). The analysis elaborates how asthmatic bodies contribute to new understandings of the role living environments play in disease trajectories and the variegated causes of air’s toxicity.

## The changing nature of asthma

The changing nature of asthma has been beautifully described in Alison Kenner’s (2018) monograph *Breathtaking: asthma care in a time of climate change* by situating knowledge and experience of asthma in wider atmospheric, ecological dynamics. Whilst focusing on individual, personal experiences of asthma, Kenner also explores ‘another form of asthma’ that is collective and public (2018, p. 4). It is this understanding of asthma, as a ‘sentinel for trouble in the air’, that inspires the analysis that follows (Ahmann and Kenner, 2020). I trace the piecing together of scientific data and evidence to highlight how the chemical ecologies that asthmatics are embedded within challenge and complicate scientific and medical models of exposure and disease. By highlighting these moments of difficulty and difference recorded in the COMEAP archive, I explore how they variously render visible the human and material relations of air pollution and the histories and political economies that enact it.

### The uncertain airs of asthma

In the 1990s, COMEAP drafted a paper with the question ‘air pollution may play a role in the induction of asthma?’ (COMEAP, 1995, p. 4). In it, the uncertain and complex causes of asthma is framed as a methodological problem that results from the way air pollution data and evidence are generated:

Where it is possible to conduct controlled experiments, as is usually the case in laboratory studies, cause and effect relationships are usually clear. The problem then is in extrapolating from these findings to effects in exposed populations. In contrast, when examining the relationship between air pollution and asthma in mobile populations using observational rather than experimental methods, cause and effect may be difficult to determine because other factors may be confounding the association […] Thus, as studies becomes more relevant to public health, they become more difficult to interpret. (COMEAP, 1995, p. 11)

The Committee resolve that although laboratory studies show the mechanisms by which air pollution could potentially play a role in the initiation of asthma, there is no ‘firm epidemiological or other evidence’ that this has occurred (COMEAP, 1995, p. 1). Observational or epidemiological studies examining the relationship between air pollution and asthma are difficult to interpret quantitatively, because of the influence of other, social, and environmental variables on results.

To address uncertainty in the role air pollution plays in asthma development, the Committee consider whether the methods used in air pollution science might be obscuring health effects. They examined how different definitions of asthma explain the causes of asthma and the various cellular, biochemical mechanisms involved in disease development. At the time of the report, knowledge of the immunopathology of asthma was changing to include other factors beyond genetics:

As the immunopathology of atopic asthma is unravelled, it is becoming possible to identify environmental factors which contribute to the development of the disease (inducers) and factors which exacerbate pre-existing disease (provoking factors) (COMEAP, 1995, p. 15).

Some individuals exhibit airway hyper reactivity in the absence of any obvious underlying tissue pathology […] there are forms of asthma with features of eosinophil [immune cells] mediated inflammation, but which are not associated with atopy (COMEAP, 1995, p. 34).

Research showing there are immune features of asthma not associated with inherited atopy or allergy provided ‘[a]n alternative mechanism of action of air pollutants’ (COMEAP, 1995, p. 29).

Unlike a clinical measure of asthma of elevated levels of antibody immunoglobulin IgE, which assumes all asthma has the same mechanistic response, the different immune features of asthma render it as heterogenous with multiple causalities (Fortun *et al.*, 2014). As a result, the committee started to explore analytical methods for separating the genetic causes of asthma from environmental ones. Ultimately, by focusing on the mechanisms that lead to the development of asthma rather than just the exacerbation of symptoms, the Committee could hypothesise that the causes of asthma might be *in* people’s breathing environments. In other words, the indeterminacy of asthma enabled the Committee to consider why some cases of asthma might be the result of ‘sensitivity’ caused by previous and repeated environmental exposures rather than genetics (Jamieson, 2017).

The report then goes on to consider the specific aspects of air quality that may play a role in the development of asthma. The Committee dedicated their attention to the chemical composition of the air people breathe and how it has changed over time. Black Carbon, an ultrafine particle from diesel emissions commonly referred to as Diesel Exhaust Particles [DEP] are high in cities where a significant proportion of the UK population live and work. The particles are also small and highly respirable. They have a large surface area, which means they can enter the lungs, be absorbed into the blood stream and travel to different organs in the body.

Examining epidemiological data of air pollution exposure in urban populations alongside this toxicological evidence of the harmful effects of diesel particles meant the Committee could identify a disease mechanism (fine particulate matter and immune cells) linked to levels of exposure (vehicle emissions in places with heavy traffic) experienced in many cities globally. Referring to the findings of these studies, they write:

These data suggest that DEP can stimulate local IgE production by nasal immune cells and hence may be significant in respiratory allergic disease. It is clear from this study that exposure level (equivalent to breathing outdoor air in Los Angeles for 24 hours) is critical and that the duration of the response is finite (COMEAP, 1995, p. 45).

Despite scientific uncertainty in the role that air pollution plays in asthma, the porosity and reactivity of bodies apparent in the documents is drawn on by scientists to explain how environments potentially modulate gene expression. The immune features of asthma that show the disease is *not* influenced by fixed genetic variables mean the ecological causes of asthma can be considered (COMEAP, 1995, p. 177). Specifically, it helped to corroborate toxicological data of diesel particle’s toxicity by translating a possible mechanism of disease causation into a *real-world* setting (‘like breathing LA’s air’).

### The particular airs of asthma

The second major output published by COMEAP on this topic, sought to explicitly test the findings of the 1995 report and provide an update on the Committee’s view of the role that air pollution may play a role in causing asthma. As the quote below shows, a correlation of increasing rates of asthma in the population and the introduction of diesel engines remained a key focus:

A possible candidate for this unknown factor [the cause of increased asthma] is diesel exhaust particulate. Sales of diesel-powered road vehicles have certainly increased in the UK over the last thirty years, and it might be reasonable to assume that exposure to particles generated by diesel engines has similarly increased (COMEAP, 2010a, p. 1).

The body with asthma becomes a signal for noticing the anthropogenic causes of pollution and urban exposure here. This serves to situate air pollution historically, geographically and as part of ‘complex socio-spatial patterns’ (Véron, 2006, p. 2093). There is a shift in focus from the disease mechanisms of asthma (the focus in 1995) towards the (changing) chemical composition of urban air, particularly in terms of the powerful processes of policy decision making around fuel use in the UK.

In Europe and the UK, the dieselisation of the car fleet by the 1990s was the outcome of industrial drivers that promoted diesel in the road fuel market, coalescing with government and tax policies that favoured diesel fuel over petrol (Fuller, 2018, pp. 134–135). The smaller particles produced by diesel emissions were at the time unregulated and not measured by government monitoring stations. The toxic property or ‘active component of air’ was therefore unknown because scientific monitoring measured the weighted mass of particles (Garnett, 2018):

It is possible that some active component of the ambient air pollution mixture has increased in these countries whilst the general level of air pollution has declined. This unknown factor might explain the high and slowly increasing prevalence of asthma. (COMEAP, 2009, p. 1)

Here, the causes of air’s toxicity and its role in disease are not perceptible in the records of air pollution data used in air quality monitoring and governance (Murphy, 2004, 2006). This is because the Committee are dealing with ‘the material effects of previous knowledge’ and decision-making, based on diesel fuel being more efficient than petrol vehicles and therefore economically beneficial (Landecker, 2015).

Attempts to identify the active or toxic chemical components of air pollution encouraged a focus inward, to the biologic mechanisms underlying the subtle interplay of diesel exhaust particles and the development of asthma. A meta-analysis of studies involving ‘human subjects’, predominantly cohort studies, was subsequently completed by the Committee, which concluded that ‘particulate matter can act as an adjuvant to sensitization to allergen’ (COMEAP, 2010b). Indeed, the review showed several potential pathways for particles to enter bodies and sensitise cellular processes and responses that lead to inflammation associated with allergy. Significantly they emphasise the dynamic biology of the lung by identifying mediators that influence gene expression (IgE antibodies) associated with inflammation and asthma:

The allergen cross-links the membrane bound IgE antibodies on sensitized mast cells, which immediately release their mediators to the surroundings. This immediate reaction can be followed by a more sustained inflammatory response, which involves cells such as eosinophils. These cells contribute significantly to the immunopathology of an allergic response. Results from experimental human and animal studies give evidence that DEP can affect several of the different stages in the allergic immune response (COMEAP, 2010b, p. 15).

The committee conclude that the toxicity of air pollution is not related to the weight of particles (i.e. a higher dose) but the number (including fine particles that are not weighed) and chemistry of particles.

Specifying the toxic component of PM2.5 by exploring possible mechanisms of immune response transforms understanding of air’s toxicity here. What is harmful about particulate pollution is contingent on specific atmospheric mixtures of particles, human activity (e.g. transportation) and actions. Previously approached as an object that is stable and comparable across cities, COMEAP’s analysis of the atmospheric chemistry of particles provides a more complex, variegated picture. They report that studies of air pollution within cities provide clearer evidence of the role of air pollution in rising cases of asthma than city level and cross-city comparative studies (COMEAP, 2010a, 2010c). Clinical evidence of the triggering of some asthma phenotypes by air pollution is highlighting changes in the air that potentially influence population and public health.

The analysis also warns that important differences in the harmful effects of air pollution are not perceptible in regulatory data (national or city-level data). Some people who have higher exposure because they live near busy roads have an asthma phenotype that is triggered by environmental exposures:

Evidence from studies on traffic-related air pollution suggests that it is possible that air pollution plays a part in the induction of asthma in some individuals who live near busy roads, particularly roads carrying high numbers of heavy goods vehicles. (COMEAP, 2010a, p. 6)

This means routinely collected air pollution data from monitoring stations can fail to capture how urban air is spatially and temporally heterogenous (and therefore also unequal). Here, the cellular level responses of the immune system make differences in the air perceptible in ways that shift the unit of analysis away from a stable body to the ongoing situated exchanges between bodies and air.

By elaborating the *particular* airs of asthma I have detailed the ways bodies are embedded in chemical ecologies, and examined how scientists handle this social. material complexity in their accounts of air pollution and health. The sensitive, responsive body draws scientists’ attention to the political and economic decisions that continue to transform the chemical composition of bodies and air over time. As a result, they broaden the causes of asthma to include social processes like policy decisions on biophysical processes at the collective level (Landecker, 2015; Neely, 2021, p. 2). My analysis therefore shows that biological differences in bodies with asthma signal material changes in air quality in ways that do not simply evidence harm but bring about new ways of knowing ‘toxic-polluted worlds’ (Calvillo, 2023).

## Asthma as a sentinel for altered bodies and atmospheres

The fact that everyone is exposed to air pollution (albeit unevenly) continues to raise significant epistemological challenges for air pollution science and policy. A control group (i.e. a group of people who are not exposed to air pollution) is commonly used in observational studies to determine whether an action (a treatment, an exposure) causes a particular health outcome. As already discussed, this is extremely complex because of the contingency of asthma on biological, environmental and biographical factors. Yet asthma rates are rising in many parts of the world, and it is now clear that air pollution is contributing to this personal and public health risk. There is therefore an urgent need to understand how and when air pollution can increase the risk of developing asthma and exacerbate its symptoms.

Wearable sensors and biomonitoring technologies are increasingly prominent in health research as a tool for monitoring and measuring risk at the individual level. In the field of air pollution science, these technologies are advancing monitoring platforms that can detect variations in exposure and provide valuable insights to inform public health and medical care (Lupton, 2018; Creager, 2018). The scientists I interviewed for this study were all involved in research using sensing technologies to measure personal air pollution exposure levels and to collect biological data relating to the health effects of exposure. These sensors were usually carried or worn on the body. In one study, a breathing sensor was worn on the skin (like a plaster) to measure respiratory rate and flow by capturing movements on the surface of abdomen (initially developed in clinical studies) as well as activity (using an accelerometer and GPS) (Drummond *et al.*, 2011). This emerging network of sensing devices monitor air pollution in the spaces and ‘rhythms’ of daily life, such as traffic, work schedules, social activities, and other urban processes (Walker *et al.*, 2022). They promise to both highlight the sources and causes of exposure as well as inform individuals on how they might reduce or avoid its risks (e.g. by changing their behaviours or using their preventive inhalers).

In the following section, I explore how these dynamic configurations of bodies and monitoring practices are using asthma’s sensitivities to make air pollution visible and actionable in the context of everyday living environments.

### The experimental airs of asthma

So far, I have detailed how asthma is an embodied, material condition that has shaped knowledge about air pollution and its harmful effects. Recent exposure experiments incorporate bodies into the sensory apparatus of scientific monitoring itself. They show people with asthma are active interpreters of air pollution, turning their bodies and experiences into vital sources for studying its impact. I refer to these airs of asthma as experimental. Researchers are increasingly interested in how bodies become immunologically sensitive to pollution, not just as a disease marker, but as part of the process of identifying harmful elements in the air, especially within real-life contexts (Gomart, 2002).

A clinician based in Delhi explained they are monitoring asthmatic bodies to understand how the disease responds to air pollution. The focus of their research is not comparing ‘a disease population’ with a ‘healthy population’. Instead, they follow the same body in day-to-day spaces of exposure and over time:

In this particular study there is basically no control group […] there is no low exposure group [instead] the idea was that we will follow the same person in three different seasons, so there are seasons like for example now it is probably intermediate exposure time for Delhi, the summers are actually low exposure, the winters are very bad […]they will have three different levels of exposure, and they will serve as their own controls in these three different seasons, and then we look at clinical outcome, their symptoms and how much medicine do they need to use for rescue, whether they have more hospitalisations or exacerbations (Interview with clinician, June 2019).

The body’s capacities and affordances are central to the research, helping this scientist study different clinical outcomes and how the interactions of bodies and air matter for health.

Similarly, a computer scientist in the UK, leading research on air pollution in India, explained how people with asthma’s bodies are sensitive enough to detect changes in their environments. Sensing networks are starting to connect chemical changes in the air with physical, biochemical responses in the body. They approach the body as ‘a spatially extensive and temporally intensive’ monitoring site, providing data on the accumulation, dispersion, and modulation of pollutants (Kelly, 2013, p. 18).

Another scientist working on the same project and responsible for analysing biomarker data describes the kinds of information they are generating about domestic lifestyles and ‘personal exposure environments’:

You are getting a phenomenal amount of data from one person over the course of a data [campaign], of so many different facets: if they’re moving inside or outside, temperature, lying down, if they’re inside, is there cooking going on next door … which is brilliant because you can really mine the situation in a real-world setting (Interview with cardiovascular scientist, November 2019).

**Figure 1.**
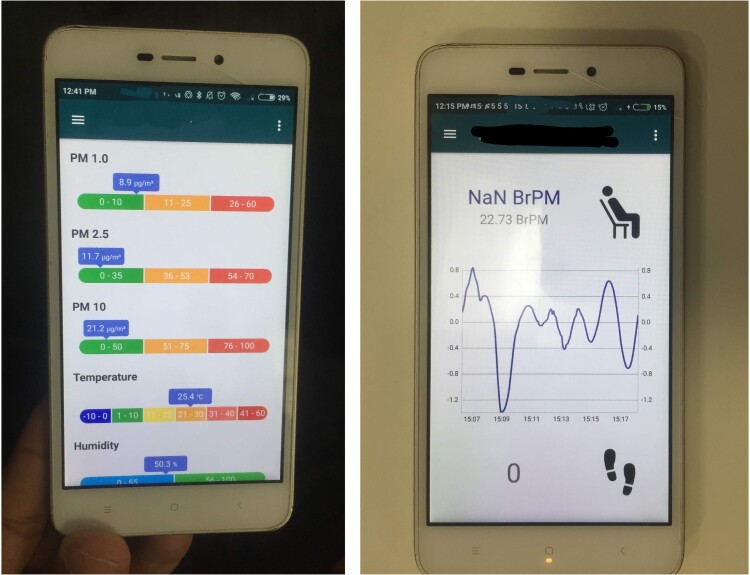
Photo of dashboard of bodily worn sensors measuring exposure and respiratory rate (author’s own).

These data are drawing attention to the rhythms and practices of daily life. They are in one sense highlighting behaviours or actions that can be modified to reduce exposure or manage (individual) health. However, they are also highlighting the richness and complexity of these processes, opening new lines of inquiry and creating opportunities for further exploration  – to ‘mine the situation’ as the scientist puts it (albeit in their research in relation to data processing) (Figure 1).

Bodily participation in air pollution sensing highlights how personal experiences reflect social, cultural material, and political aspects of living with asthma. Bodies are ‘the bearers of information’ of the air’s changing state, but there is rarely a single source or responsible agent for pollution and asthma (Gramaglia and Mélard, 2019, p. 816). In this set up, the asthmatic body becomes an index of air pollution, offering insight into the sources of pollution and the worlds that produce it (Frost, 2021; Meloni *et al*., 2021).

These sensing networks therefore do not just expose the presence of air pollution or environmental risk and injustice. They also generate knowledge and insight into ways of living with air pollution and point to a complex, multifaceted source of harm. It is this kind of knowledge that is relevant for preventing harm. For instance, how people use the sensor or sense with their bodies highlights when and where air pollution is a particular concern or problem. The non-use of an inhaler or removal of a sensor points to the social and cultural experiences of disease, which need to be considered when developing treatments and forms of care. These sensing and knowing bodies reveal exposure patterns but also how pollution impacts individuals in ways that are situated, accumulative and enduring.

Despite this emerging engagement with bodily data, the impact of current exposure research tends to be narrowly framed. Personal monitoring studies while valuable for precision medicine (McCarron *et al.*, 2024) are typically presented in terms of individual health management rather than broader societal implications. An exposure scientist working on a range of sensing studies in the UK and India said participants are treated as passive actors in research and how data might be used and applied in practice is not usually considered:

in biomedical research you don’t tell the participants anything. I remember having a debate with a clinician and saying we should give them [patients] back exposure results with high levels they could do something about […] (Interview with exposure scientist, January 2020).

Other researchers also reported difficulties in encouraging participation due to the effort required for using sensors, as well as cultural factors that discourage engagement (Public health fieldworker interview, June 2019; see also Senanayake, 2021). In the research discussed by the people I interviewed, participants apparently misused or removed the sensors during specific activities, such as playing or going to school. These challenges highlight a likely discrepancy between personal exposure data and an individualised approach to managing risk which assumes health is a choice (Braun, 2007).

The work and actions of sensing bodies  – through the wearing of sensors, collection of biomarker data, or through listening to patient’s experiences  – reveals the complexities of human entanglements with their atmospheric environments. I have emphasised here how this practice of sensing is responsive and involves the active mediation of bodies in knowledge production. This approach is bringing into view the material and historical contexts of exposure and health. Sensory experiences, then, are an effective medium for understanding the human-non-human-relations of pollution, and the powerful systems and political economies that enact it. Yet, by limiting the ways bodies participate in air pollution research, and treating them as simply a passive site of data generation, means critical knowledge for responding to the risks and harms of air pollution are being disregarded (Savranksy, 2019).

## Conclusions

How the external environment becomes part of the body and how the body is part of its own remaking challenges science and policy attempts to know and govern air pollution. Air pollution science is turning to the body with the goal of generating more granular understandings of exposure and disease. However, viewing the body through a scientific and medical framing is constraining efforts to develop the kinds of knowledge and responses this environmental health concern demands. It is reinforcing Western liberal ideals of individual responsibility and overlooking the chemical ecologies that bodies are embedded in, which shape exposure and disease in significant ways.

Contributing to literature that treats pollution as an assemblage of human-non-human relations, this article explores how scientific efforts to understand the role air pollution plays in asthma requires a more expansive, active treatment of bodies in research. I extend concepts of sensing in STS that have focussed on the subjective, embodied experiences of pollution (Shapiro, 2015; Jarowski, 2022). This can inadvertently reinforce scientific understandings of the body that separate subjective experiences from scientific claims about air pollution and its harmful effects. Instead, informed by recent social science engagement with environmental epigenetics, I detail how bodies are responsive to their environments and therefore deeply entangled in data and evidence about air's toxicity (Landecker, 2015, 2016; Frost, 2021; Meloni *et al.*, 2021). If bodies are treated as active participants in knowledge about air pollution, then what kinds of research are undertaken can also be re-considered, moving beyond the pre-determined foci of science and policy.

The empirical analysis traces how the material and fleshy experiences of bodies have shaped knowledge and evidence about health in air pollution science. I detail how scientists have engaged with questions of uncertainty and indeterminacy in the role air pollution plays in asthma in ways that highlight the historical, socio-political and everyday practices that produce pollution exposure. I show how in the documentation of the COMEAP archive, science and policy discussions about the effects of air pollution on health incorporate the body in the production of knowledge. How bodies become sensitive to air pollution over time through bio-chemical alterations suggests it is in certain contexts and life-stages that exposure can be harmful. This sensing body is explored further in my analysis of scientists’ accounts of doing contemporary exposure research. I examine how the sensing body is also responsive and knowing, becoming part of the monitoring apparatus of science itself – drawing connections between individual experiences and the broader ecologies of air pollution. However, at present, this work is translating research into actions and recommendations that separate the known body from its everyday experiences, thereby limiting the generation of more critical knowledge that are necessary for dealing with air pollution.

Revisiting the two questions posed at the outset of this article: *What is the role of the body in research about air pollution and health? What are the possibilities (and limitations) of extending sensing to bodies for air pollution knowledge and politics?* In the notes and data sets of the COMEAP archive, the body is materialised as a sensorium of chemical reactions, responses and alterations. I detail how the biological status of ‘sensitivity’ meant the body could be approached in dynamic relation with its environments, which ultimately helped scientists connect the chemical composition of particles in the UK to the history and political economy of decision-making. Turning to contemporary research, through bodily-worn monitors, scientists are starting to use the body’s surfaces, structure and biochemistry to generate data that reflects the dynamic exchanges between bodies and air. More specifically, knowledge of the heterogeneity of asthma and the various ways it presents in people’s day to day lives is being used to draw attention to the spaces, times and social practices of exposure. As well as more granular data, this research is making visible social and spatial differences in air pollution.

To illustrate this argument, I return to the third question this article opened with: *How may they [sensing bodies] offer ways of knowing, responding, and living with air pollution in the context of multiple and accumulating exposures?* My analysis emphasises how the body is not simply a new scale for evidencing health effects but a starting point for bringing lived worlds into focus, including the spaces (homes, streets), actions (cooking, commuting) and mutual exchanges of bodies and air (moving the body differently in response to breathing air pollution). Participatory approaches to sensing and research are producing novel insights into how people live with and navigate air pollution in material and embodied ways. These are currently being overlooked in the generation of evidence and responses to air pollution by scientists, however. The ways people use and engage with sensors, for instance, is often interpreted as troubling data quality or disrupting data collection in a clinical trial. By approaching sensing bodies as agents in knowledge of air pollution it is possible to rethink and move beyond this official line of improving health outcomes through better data (Savranksy, 2019). I suggest that by centring the sensing body scientific studies might be better positioned to generate knowledge that is relevant for informing practical actions that could ultimately help prevent (rather than just treat) harm.

This article contributes to on-going attempts in STS and the social sciences to know and respond to pollution and its embodied effects in other ways than monitoring and measurement (Fortun, 2014; Liboiron *et al.*, 2018). Focusing on the epigenetic relationship between air pollution and asthma, I expand concepts of sensing in STS to show how the sensory affordances of bodies are entangled in scientific ways of knowing rather than necessarily distinct or separate. It therefore advocates for expanding the concept of sensing to include ‘knowing bodies’. In doing so, it also provides alternative starting points for re-thinking participation in research around issues like air pollution by emphasising the human-non-human entanglements of living with it. Participation in health research need not reduce participants to atomised individuals that end up being responsible for their own health (although this remains a risk) (Prainsack, 2014). Grounding science in the more critical forms of knowledge that are emerging in air pollution science would not only foster a deeper sense of responsibility for its complex causes but also help inform actions to prevent harm.

## Notes

1 The COMEAP government webpage and the National Archives website provide public access to the meeting minutes, reports and statements that I use in this article: https://www.gov.uk/government/groups/committee-on-the-medical-effects-of-air-pollutants-comeap; https://webarchive.nationalarchives.gov.uk/ukgwa/20140505105518/http://www.comeap.org.uk/documents/minutes-and-agendas.
